# Clinical Management of Filovirus-Infected Patients

**DOI:** 10.3390/v4091668

**Published:** 2012-09-21

**Authors:** Danielle V. Clark, Peter B. Jahrling, James V. Lawler

**Affiliations:** 1 NIH/NIAID Integrated Research Facility, Fort Detrick Maryland, USA; Email: Danielle.clark.ctr@med.navy.mil; jahrlingp@niaid.nih.gov; 2 Naval Medical Research Center - Frederick, Frederick Maryland, USA; Email: james.lawler2@med.navy.mil

**Keywords:** Filoviruses, Ebola, Marburg, Clinical management, Treatment, Outbreak

## Abstract

Filovirus infection presents many unique challenges to patient management. Currently no approved treatments are available, and the recommendations for supportive care are not evidence based. The austere clinical settings in which patients often present and the sporadic and at times explosive nature of filovirus outbreaks have effectively limited the information available to evaluate potential management strategies. This review will summarize the management approaches used in filovirus outbreaks and provide recommendations for collecting the information necessary for evaluating and potentially improving patient outcomes in the future.

## 1. Introduction

In humans, filovirus infection results in a spectrum of illness, but most recognized infections present as severe acute febrile illness with a high proportion of fatalities. The typical clinical presentation consists of acute onset of a non-specific febrile illness, including chills, headache, myalgia, nausea/vomiting, and diarrhea. A faint rash develops in 25-52% of patients in the first week [1]. Minor hemorrhagic manifestations are noted in some patients after a few days of illness (e.g., conjunctival hemorrhage, petechiae, ecchymoses, bleeding from puncture sites). In many instances, a biphasic pattern can occur, with a brief remission followed by a recurrence of fever and more severe late stage disease. In later stages of the severe forms of illness, patients demonstrate hypotension, shock, mucosal hemorrhages (typically from the gastrointestinal tract) and multi-organ system (particularly renal) failure [2,3]. Although autopsies demonstrate multifocal necrosis, they have generally not identified specific pathological lesions responsible for death [4]. Nevertheless, severe cases are frequently fatal, with ultimate demise attributed to the systemic effects of a septic shock-like syndrome. No licensed or approved specific medical countermeasures exist, making supportive care the cornerstone of patient management. 

Provision of supportive care, as well as detailed documentation of the care given, is challenging in austere settings in which most large filovirus outbreaks occur. Laboratory equipment, supplies (particularly personal protective equipment), and adequate infrastructure are often lacking. Healthcare worker attrition and abandonment of suspected cases can result from actual infection or fear of infection, perceived inability to affect the clinical course, and community stigmatism. In some outbreaks, communities hide sick patients and dead bodies [5], thus, forcing efforts to focus on community surveillance. 

In this review, we summarize the management approaches documented in published outbreak reports, conference proceedings, journal articles, and a recently published compendium of the filovirus literature [6]. We highlight gaps in knowledge of clinical management of filovirus patients and areas where our current outbreak response strategies could be enhanced to improve patient care. 

## 2. Results and Discussion

There have been 34 recognized filovirus outbreaks, including sporadic cases and laboratory accidents, since 1967. Perhaps not surprisingly, the most detailed information on the clinical management of filovirus-infected patients came from outbreaks or cases occurring in developed countries. In general, the clinical care provided to filovirus patients has varied widely, primarily due to resource constraints of many outbreak settings. 

### 2.1. Supportive Care Provided in Filovirus Outbreaks

The clinical presentation of filovirus-infected patients is difficult to distinguish from other infections, especially early in the clinical course. Given that laboratory diagnostic capabilities were often limited in outbreak settings, patient care typically followed the routine approach to severe febrile illness in the tropics, beginning with antibiotics and antimalarial drugs [Table 1]. In many cases, antibiotics were also used to prevent and/or treat secondary bacterial infections. Acyclovir was used in one patient in the 1976 Zaire outbreak, and ribavirin was given to one patient in Russia. No other employments of antiviral drugs were documented. Analgesics, antipyretics, and antiemetic drugs were typically available and administered as needed. Unfortunately, many patients did not receive any further care. Other symptomatic treatments occasionally available included antidiarrheal drugs, sedatives, and antipsychotic drugs to reduce anxiety and agitation. 

Oral rehydration was routinely encouraged, but at times not administered partially due to the close proximity required to prop up a severely ill patient so they could drink [7]. Oral rehydration was typically preferred to administration of IV fluids, partially due to the perceived risk of transmission associated with the use of needles as well as resource constraints. Fluid and electrolyte monitoring and supplementation were universally applied to patients in well-equipped hospitals, but these measures were not routinely available during most outbreaks. Intravenous (IV) fluids were first used in a developing country setting in the 1976 outbreak in Zaire for the treatment of three nuns [8]. The first large filovirus outbreak in Africa in which IV fluids were routinely used was in Kikwit, Zaire (now Democratic Republic of the Congo) in 1995, but such fluids were used only in the last few weeks of the outbreak (approximately 25 patients) [9]. Hartmann’s solution and lactated Ringer’s solution have been used, but in most reports, the type of IV fluid administered was not specified. Pressor agents (epinephrine) were occasionally administered to prevent shock.

Various blood products, clotting factors, inhibitors of fibrinolysis (ε-aminocaproic acid) and regulators of coagulation were administered to counteract hemorrhage. Transfusion of blood components included whole blood, packed red blood cells, fresh frozen plasma, and platelets. Clotting factors and other regulators of coagulation administered included fibrinogen, and prothrombin, proconvertin, Stuart-factor and antihemophilic globulin B, and vitamin K. In contrast, anticoagulants (heparin) and rheologic agents (pentoxyfylline) were given in some patients to prevent thrombosis and disseminated intravascular coagulation. 

Attempts at passive immunotherapy included use of convalescent blood, plasma or serum. Convalescent sera was first administered to four patients during the 1967 Marburg virus disease outbreak in Frankfurt, West Germany (now Germany) [10] and in Belgrade, Yugoslavia (now Serbia) [11]. Gamma globulin infusions were used in 1 patient in Yambuku, Zaire [8] and interferon in one patient in the UK in 1976 [9,10,12,13] and one patient in Russia in 2004 [14]. Polyoxidonium, an immunomodulator, was also given to the patient in Russia [14].

Support for organ failure, including dialysis, hemofiltration, intubation, and mechanical ventilation was only available for a small number of patients in developed-country settings.

### 2.2. Treatment Efficacy

None of the supportive care strategies used in the field were prospectively evaluated to determine treatment efficacy. Transfusion of blood from convalescent patients was highlighted as potentially useful in Kikwit, Zaire when only one of eight patients receiving a transfusion died [15]. However, these patients received substantially better care than those in the early stages of the epidemic, and treatment started relatively late in the disease course, indicating that selection bias may have contributed to the positive results [15,16]. Ebola convalescent serum had been administered to three additional patients in two separate outbreaks, all of which survived [6,12]. Five patients in four separate outbreaks received IV heparin; two of the five patients survived [8,14,17,18]. Dehydration was noted in several outbreaks as potentially contributing to the high mortality, but as with other therapies, the effect of IV fluid administration has not been rigorously evaluated. 

**Table 1:** Clinical Management Approaches Used in Filovirus Disease Outbreaks.

Year	Virus	Location(s)	No. Cases, No. Deaths(Percent)	Clinical Management	Ref*
1967	MARV	Marburg & Frankfurt, West Germany (Germany); Belgrade, Yugoslavia(Serbia)	31, 7 (22.6%)	♦ Antibiotics (tetracycline, chloramphenicol, penicillin, oxytetracycline, cephalothin, streptomycin, bleomycin, nystatin) used prophylactically ♦ Antipyretic (dipyrone) ♦ Cardiac glycosides (ouabain, digitalis, digitoxin, strophanthin) ♦ Steroids (prednisone [2 patients], nandrolone phenylpropionate) ♦ Electrolyte and fluid supplementation, low sodium human albumin infused (hypoproteinaemia) ♦ Renal dysfunction treated with peritoneal dialysis, mannitol, furosemide ♦ Fresh blood platelet concentrates, fibrinogen, ε-aminocaproic acid, vitamin K, and PPSB ♦ Convalescent sera (4 patients)	[10,11,19,20,21]
1975	MARV	Rhodesia (Zimbabwe), transfer to Johannesburg, South Africa	3, 1 (33.3%)	♦ Antibiotics (ampicillin, chloramphenicol) and antimalarials (chloroquine) used initially ♦ Analgesics (hydroxyzine) pentazocine ♦ Parenteral fluids (~4 l/24 h) and electrolyte monitoring ♦ Renal failure treated with peritoneal dialysis (1 patient) ♦ Fresh blood, fresh-frozen plasma, and platelet infusions for hemorrhagic diathesis ♦ Prophylactic heparin (2000 U loading, 10,000 U infusion/24 h, PTT guided, 2 patients) ♦ Plasma from a Lassa fever survivor (suspected Lassa fever, IV, 2 patients)	[17,22]
1976	EBOV	Yambuku, Zaire (DRC)	318, 280 (88.1%)	♦ Unspecified antibiotics and antimalarials ♦ Antipyretic (aspirin) and anti-diarrheal drugs Clinical management for 3 nuns (all 3 died) ♦ Antibiotics, antiviral (acyclovir ,1 patient) ♦ Aspirin, anti-diarrheal drugs, and diazepam (1 patient) ♦ IV fluids ♦ Blood transfusions, vitamin K, gamma globulin (1 patient) ♦ Prophylactic heparin (2,000 U loading, 16,000 - 30,000 U/24 h, 1 patient) ♦ Hydrocortisone and epinephrine (1 patient), digitalis (1 patient) ♦ Marburg convalescent plasma (1 patient)	[8,22,23,24,25]
1976	SUDV	Nzara, Sudan (South Sudan)	284, 151 (53.2%)	♦ Unspecified antibiotics and antimalarials (injections) ♦ Fluid and electrolyte balance (late stages of outbreak)	[26,27,28]
1976	SUDV (maybe EBOV?)	U.K.	1, 0 (0%)	♦ IM Interferon (3million U/12 h for 14 days, initiated 20h post onset) ♦ Antinausea (metoclopramide ),antidiarrheals ( diphenoxylate hydrochloride and atropine sulfate) antifungal (amphotericin B lozenges) ♦ Fluid replacement (Hartmann’s solution) ♦ Ebola convalescent serum (450 ml infusion over 4 h, 47 h post onset, plus 330 ml on day 6)	[12,13]
1977	EBOV	Tandala, Zaire (DRC)	1, 1 (100%)	♦ No information found on clinical management	[29]
1979	SUDV	Nzara, Sudan (South Sudan)	34, 22 (64.7%)	♦ No information found on clinical management	[30,31,32]
1980	MARV	Kenya	2, 1 (50%)	♦ Unspecified antibiotics and antimalarials ♦ Rehydration (unspecified route)	[33]
1987	RAVV	Kenya	1, 1 (100%)	♦ Antibiotics (chloramphenicol and cloxacillin) and antimalarials (chloroquine phosphate prophylaxis, pyrimethamine sulfadoxine, amodiaquine, sulfalene) ♦ High dose steroids ♦ Heparin, fresh plasma, blood transfusion ♦ Dialysis (2x)	[18]
1988	MARV	Koltsovo, Soviet Union (Russia)	1-2, 1-2 (50%-100%)	♦ No information found on clinical management	[34,35]
1990	MARV	Koltsovo, Soviet Union(Russia)	1, 0 (0%)	♦ Extracorporeal hemo-sorbent and hemodialysis	[36,37]
1994	TAFV	Côte d'Ivoire	1, 0 (0%)	♦ Unspecified antibiotics, antimalarials (halofantrine and IV quinine) ♦ Lactated Ringer’s solution (dehydration) ♦ Repatriated to Switzerland (day 7), treated for suspected sepsis, leptospirosis or rickettsia	[38]
1994-95	EBOV	Mékouka, Gabon	52, 32 (61.5%)	♦ No information found on clinical management	[39,40,41,42,43]
1995	EBOV	Kikwit, Zaire (DRC)	317, 245 (77.3%)	Early in VHF outbreak: [Minimal care provided, suspected cases abandoned initially] ♦ Antibiotics and antimalarials (chloramphenicol, cotrimoxazole, tetracycline, nalidixic acid) ♦ Acetaminophen, metoclopramide, haloperidol (nausea, hiccups), diazepam (agitation, epilepsy) ♦ Oral treatments only Clinical management in the last weeks (25 patients, 14 deaths (56%)) ♦ Protein-rich liquid ♦ IV fluid infusion (dehydration) ♦ Convalescent blood transfusion (8 patients)	[2,3,9,15,16,44]
1996	EBOV	Mayibout (2), Gabon (2 secondary cases in South Africa)	31, 21 (67.7%)	♦ Clinical Management in South Africa (2 patients, 1 death (50%)) ♦ Unspecified antibiotics ♦ Dialysis, intubation and ventilation, parenteral nutrition ♦ Pulmonary artery flotation catheter (hypotension) ♦ Epinephrine infusion (titrated to systemic vascular resistance and left ventricular stroke work index), amiodarone (tachyarrhythmias) ♦ Patient 2: (Retrospective dx) 250 mg IV hydrocortisone (suspected autoimmune condition) ♦ Clinical Management in Gabon ♦ IV fluids reportedly used ♦ Symptomatic treatment and palliative care	[9,45,46]
1996	EBOV	Russia	1, 1 (100%)	♦ No information found on clinical management	[47]
1996-97	EBOV	Booué, Gabon	62, 46 (74.2%)	♦ Antibiotics (ampicillin, nifuroxazide) ♦ Acetaminophen ♦ Ringer-lactate infusion	[48]
1998-2000	MARVand RAVV	Durba and Watsa, DRC	154, 128 (83.1%)	♦ Unspecified antibiotics and antimalarials ♦ Acetaminophen, antiemetics (chlorpromazine, metoclopramide), antacid (aluminum hydroxide) ♦ IV fluids available (dehydration)	[9,49,50]
2000-01	SUDV	Gulu and Masindi, Uganda	425, 224 (52.7%)	Clinical management in Masindi (76% case-fatality): ♦ Analgesics and sedatives ♦ Oral rehydration [insufficient assistance noted], IV fluids occasionally	[7]
2001-02	EBOV	Mékambo, Gabon, Mbomo, Kéllé, RC	124, 97 (78.2%)	♦ Oral rehydration, IV fluids if necessary ♦ Acetaminophen ♦ Nutritional support ♦ Blood transfusion (shock) ♦ IV quinine (isolated case in Franceville)	[51,52,53,54,55,56,57,58,59,60,61,62]
2002	EBOV	Gabon and RC	11, 10 (90.9%)	♦ No information found on clinical management	[53,59,63]
2002-03	EBOV	Kéllé and Mbomo, RC	143, 128 (90%)	♦ No information found on clinical management	[58,64,65,66,67,68,69,70,71]
2003-04	EBOV	Mbomo, RC	35, 29 (82.9%)	♦ Symptomatic oral treatments for dehydration and fever	[72,73]
2004	EBOV	Koltsovo, Russia	1, 1 (100%)	♦ Antibiotic prophylaxis (day 7: IV ciprofloxacin 200mg 2x/d, change to IV perfloxacin 400mg 2x/d) ♦ Ribavirin (600mg/d for 1^st^ 6 days) ♦ Topical hydrocortisone (rash), dexamethasone (16mg/d IV, starting on day 7) ♦ EBOV hyperimmune equine serum IM ≈4 hours post exposure (12 ml, titer 8,912) ♦ Alpha-interferon (1 million U 2x/d for 1^st^ 5 days), polyoxidonium (0.006g/d IM), tilorone (0.125g/d starting on day 6) ♦ Low-molecular weight heparin (fraxiparin 2500 U 4x/d), rheologic agents (reopolyglutin 400 ml 3x/d, pentoxifylline 5ml 3x/d) ♦ Repeated plasmapheresis (days 7-10) ♦ IV fluids (Ringers solution 400 ml), electrolytes, glucose, insulin ♦ Fresh-frozen plasma (up to half the total plasma volume in body)	[6,14]
2004	SUDV	Yambio, Sudan(South Sudan)	17, 7 (41.2%)	♦ No information found on clinical management	[74,75,76,77]
2004-05	MARV	Uíge, Angola	374, 329 (88.0%)	♦ No care given initially ♦ Antibiotics (cortrimoxazole) and antimalarials (artemisinin combination therapy) ♦ Analgesics (acetaminophen), sedatives, antiemetics (promethazine), cimetidine (dyspepsia) ♦ Oral rehydration only in first 2 months, IV fluids after month 3	[5,78]
2005	EBOV	Etoumbi, RC	11, 9 (81.8%)	♦ No information found on clinical management	[79,80]
2007	MARV and RAVV	Kamwenge, Uganda	4, 2 (50.0%)	♦ No information found on clinical management	[81,82,83,84]
2007	EBOV	Luebo, DRC	264, 187 (70.8%)	♦ No information found on clinical management	[85,86,87,88]
2007	BDBV	Bundibugyo, Uganda	131, 42 (37%)	♦ Unspecified antibiotics and antimalarials if indicated ♦ Antipyretics ♦ Fluid monitoring, oral rehydration when possible, IV fluids otherwise	[89,90]
2008^ŧ^	MARV	Uganda (transferred to USA)	1, 0 (0%)	♦ Malaria prophylaxis (atovaquone-proguanil) ♦ Ciprofloxacin (2 doses, diarrhea), doxycycline (suspected leptospirosis) ♦ Antiemetics ♦ IV fluids ♦ Cholecystectomy (acalculous cholecystitis) ♦ Blood transfusion after hospital discharge (persistent anemia)	[91]
2008	MARV	Uganda (transferred to Netherlands)	1, 1 (100%)	♦ Ceftriaxone (IV, suspected typhoid fever) ♦ Fluid monitoring, norepinephrine and epinephrine ♦ Fresh frozen plasma (40U, 300 ml each), thrombocytes (6U), packed RBCs (9U, 270ml each) ♦ Mechanical ventilation (respiratory failure) ♦ Continuous venovenous hemofiltration w/ molecular absorbent recirculation system (MARS) ♦ IV glucose and insulin, bicarbonate, and high dose calcium (electrolyte imbalance) ♦ IV mannitol and hypertonic saline (cerebral edema)	[92]
2008-09	EBOV	Mweka and Luebo, DRC	32, 15 (46.9%)	♦ No information found on clinical management	[86,93,94,95]
2011	SUDV	Uganda	1, 1 (100%)	♦ No information found on clinical management	[96]

*The references refer to articles where information on management was obtained. If no information on management was found, the references refer to articles that were reviewed. ŧ Patient was retrospectively diagnosed with MARV by repeat convalescent serology and detection of MARV RNA in archived convalescent serum. Notes: The most current filovirus taxonomy and nomenclature is used in the table [97,98,99]. The recent outbreaks in Uganda (July 2012) and DRC (August 2012) are not included as no information is yet available on patient care. MARV=Marburg virus, RAVV=Ravn virus, EBOV=Ebola virus, SUDV=Sudan virus, TAFV= Taï Forest virus, BDBV=Bundibugyo virus, PPSB=prothrombin, proconvertin, Stuart-factor and antihaemophilic globulin B, PTT=partial thromboplastin time, IV=intravenous, IM=intramuscular, RBC=red blood cells, DRC=Democratic Republic of the Congo, RC=Republic of the Congo, USA=United States of America

### 2.3. Clinical Documentation and Dissemination

No information on clinical management was found for 13 of the 34 outbreaks. Of those outbreaks that documented and published information on patient management, the description of the exact treatment regimen(s) was often vague. Patient management reportedly improved over time in many outbreaks [9,26,78]. In particular, Guimard et al. reported the patient care separately by phase of the outbreak in Kikwit, Zaire [9] in order to reflect this change. 

## 3. Experimental Section

We searched PubMed for articles relating to each filovirus disease outbreak individually, as well as for articles on treatment for filovirus infections (“Ebola AND treatment”, “Marburg AND treatment”, and “filovirus AND treatment”, species filter: Humans). We obtained additional references from a recent compendium of filovirus literature [6], as well as a recent review of the clinical and laboratory features of filovirus infection [1]. In total, we reviewed over 200 journal articles, outbreak reports, and conference proceedings [100,101,102,103,104]. Articles in German or French were screened using an online translation tool (Google Translate); a native speaker screened two articles in Russian [14,47]. 

## 4. Recommendations and Conclusions

Although there have been many advances in our understanding of the pathophysiology of filovirus infections and progress towards development of vaccines and therapeutics, insufficient attention has been paid to evaluating and improving the supportive care of filovirus patients. The contrast in the case-fatality observed in patients with access to modern hospital facilities versus those in resource-constrained settings has been previously noted, causing many to speculate that substantial improvements in survival could be made through enhanced supportive care [105,106]. Several management strategies have been attempted in outbreak settings, as summarized in this review, but none of the strategies have been implemented or documented to permit evaluation of the treatment efficacy. As noted by Colebunders et al., improvements in clinical documentation are required for basic evaluation of supportive treatment [50]. In the absence of a clinical trial, accurate collection and timely publication of observational data on patient demographics, clinical signs and symptoms, treatment, and clinical outcome are crucial. 

Barring the development of an effective drug or vaccine, clearly the most critical need in the improvement of filovirus infection management are scientifically collected data to validate clinical interventions and provide a foundation for evidence-based guidance. As the pathologic process of filovirus infection resembles that of severe sepsis and septic shock, a reasonable management strategy is aggressive supportive care using standard protocols for severe sepsis [105,106,107]. The efficacy of early goal-directed therapy (EGDT) and the related Surviving Sepsis Campaign management protocols has been well established in highly-resourced medical facilities across a broad range of common pathogens [108,109,110,111]. Nevertheless, the applicability of these approaches has yet to be tested or validated with more exotic diseases (including filovirus infections) commonly presenting as severe sepsis in tropical or under-developed regions. In addition, although theoretically possible, such approaches have not been proven to be feasible in medical facilities in resource-limited settings [112,113]. 

EGDT requires early fluid resuscitation within the first 6 hours of presentation; most algorithms include an initial bolus of 20-40 ml/kg of crystalloid solution followed by frequent volume boluses sufficient to meet specific goal targets with respect to indices such as central venous pressure (CVP), mean arterial pressure, and mixed venous oxygen saturation (SvO_2_). The rationale for this approach in severely septic patients was summarized in a meta-analysis [114]. Significant mortality benefit was seen in studies where hemodynamic optimization in critically ill patients was initiated before the development of organ failure when compared to those studies that achieved optimization after the occurrence of organ failure. Notably, fluid resuscitation alone can help to reduce the global tissue hypoxia that is central to the development of multi-organ dysfunction by increasing the cardiac output and improving oxygen delivery to the tissues [115]. While fluid resuscitation alone, as dictated by EGDT, may have benefit, the Surviving Sepsis Campaign created management protocols that are a group of interventions related to a disease that, when implemented together, result in better outcomes than when implemented individually [116]. The synergistic effect of appropriate therapies given together at the appropriate time may alter the pathophysiologic course of disease enough to improve the outcome. Finally, it should be noted that some experts argue that aggressive fluid resuscitation generally attributed to EGDT are actually contraindicated in the management of filoviruses, given the associated endothelial dysfunction and theoretical risk of pulmonary edema. 

Evaluation of enhanced supportive care and development of tailored protocols for management of filovirus infections should be priorities in the response to future outbreaks. The challenges inherent in this task are considerable, but not insurmountable. Strategies for conducting clinical research in filovirus disease outbreak settings have been proposed, including establishment of a collaborative platform for development and approval of research protocols in advance of an outbreak [106,107]. Furthermore, the research capacity required to conduct clinical trials to evaluate sepsis management strategies in austere settings, namely the availability of institutional review boards, community outreach programs, trained personnel, clinical supplies, and laboratory diagnostics, could be adapted to filovirus disease outbreak settings. Provision of enhanced supportive care, accurate clinical documentation, and rigorous evaluation of innovative treatment strategies may improve patient outcomes in future filovirus disease outbreaks.
